# A QUALITATIVE EXPLORATION OF A PSYCHOSOCIAL INTERVENTION FOR LONELINESS AND SOCIAL ISOLATION IN OLDER ADULTS

**DOI:** 10.1093/geroni/igae098.4104

**Published:** 2024-12-31

**Authors:** Max Zubatsky, Marla Berg-Weger, Dimitra Tziarli, Rachel Livingston, Jen Lauck

**Affiliations:** Saint Louis University, St. Louis, Missouri, United States; Saint Louis University, St. Louis, Missouri, United States; Saint Louis University, St. Louis, Missouri, United States; Saint Louis University, St. Louis, Missouri, United States; Saint Louis University, St. Louis, Missouri, United States

## Abstract

Loneliness and social isolation is a public health epidemic in older adults. Although providers are exploring more psychosocial interventions to help curb this trend in the community and healthcare settings, very few evidence-based approaches have been able to deliver effective interventions and strategies for this population. Circle of Friends (CoF) is an emerging global intervention that has been implemented in a variety of settings for older adult socialization and improving overall mood. The CoF group consists of weekly one hour sessions over 12 weeks, targeting different themes around health and lifestyle, arts and hobbies, and theraeutic writting and activities. The purpose of this study is to explore the experiences of participants in their involvement of Circle of Friends from three different group settings (university, virtual, and primare care). A focus group format helped collect qualitative themes from 11 participants across three completed groups between 2022-2024. Overall, a consensus of main themes were found across all three groups regarding access to a social group, motivation to improve other health and lifestyle activities, and better coordination with other providers and resources in their area. Over 50% of the reports from these focus groups mentioned the lack of any social group options or resources given by their healthcare team (Geriatrics or Primary Care). This study highlights not only the impact that a psychosocial group has for older adult patients, but the need for more older adult group options in integrated care and community settings in the older adult population.

